# The roles of XJ13 and XJ44-specific mutations within the Candid #1 GPC in Junin virus attenuation

**DOI:** 10.3389/fimmu.2023.1172792

**Published:** 2023-06-02

**Authors:** John Tyler Manning, Junki Maruyama, Timothy Wanninger, Rachel A. Reyna, Heather L. Stevenson, Bi-Hung Peng, Emily K. Mantlo, Cheng Huang, Slobodan Paessler

**Affiliations:** Department of Pathology, The University of Texas Medical Branch, Galveston, TX, United States

**Keywords:** Candid #1 vaccine, Junin virus, neurovirulence, vaccine safety, arenavirus diseases

## Abstract

Junin virus (JUNV) is a member of the Arenaviridae family of viruses and is the pathogen responsible for causing Argentine hemorrhagic fever, a potentially lethal disease endemic to Argentina. A live attenuated vaccine for human use, called Candid#1, is approved only in Argentina. Candid#1 vaccine strain of Junin virus was obtained through serial passage in mouse brain tissues followed by passage in Fetal Rhesus macaque lung fibroblast (FRhL) cells. Previously, the mutations responsible for attenuation of this virus in Guinea pigs were mapped in the gene encoding for glycoprotein precursor (GPC) protein. The resulting Candid#1 glycoprotein complex has been shown to cause endoplasmic reticulum (ER) stress in vitro resulting in the degradation of the GPC. To evaluate the attenuating properties of specific mutations within GPC, we created recombinant viruses expressing GPC mutations specific to key Candid#1 passages and evaluated their pathogenicity in our outbred Hartley guinea pig model of Argentine hemorrhagic fever. Here, we provide evidence that early mutations in GPC obtained through serial passaging attenuate the visceral disease and increase immunogenicity in guinea pigs. Specific mutations acquired prior to the 13th mouse brain passage (XJ13) are responsible for attenuation of the visceral disease while having no impact on the neurovirulence of Junin virus. Additionally, our findings demonstrate that the mutation within an N-linked glycosylation motif, acquired prior to the 44th mouse brain passage (XJ44), is unstable but necessary for complete attenuation and enhanced immunogenicity of Candid#1 vaccine strain. The highly conserved N-linked glycosylation profiles of arenavirus glycoproteins could therefore be viable targets for designing attenuating viruses for vaccine development against other arenavirus-associated illnesses.

## 1 Introduction

Junin virus (JUNV) is the human pathogen responsible for Argentine hemorrhagic fever (AHF), which can potentially result in lethal illness when misdiagnosed and/or untreated. The illness can typically be characterized by both hemorrhagic manifestations and involvement of the central nervous system (CNS) (1). In severe AHF, patients present with neurological manifestations that can include ataxia, seizures, muscular hypotonia, areflexia, and coma (2). Thrombocytopenia and leukopenia are detectable during the first two weeks of infection. Hemorrhagic manifestations typically involve petechiae in the mouth and bleeding of the gums (3). Mortality rates can be as high as 30% in untreated cases. The outbred Hartley guinea pig model of AHF reproduces most clinical AHF symptoms and findings, including fever, leukopenia, thrombocytopenia, hemorrhagic manifestations, liver enzyme elevations in plasma and death around 14 days post-challenge. During this time, the guinea pigs do not develop detectable levels of virus-specific antibodies (4–6). Treatment of guinea pigs with immune serum, while very efficacious against acute disease, sometimes results in the development of a late neurological syndrome with rear-limb paralysis (7).

Arenavirus genomes consist of two single-stranded, negative-sense RNA segments, termed the small (S) and large (L) segment. Each RNA segment contains two genes organized in an ambisense coding strategy. The S segment (~3.4 kb) encodes both the nucleoprotein (NP) and the glycoprotein precursor (GPC) genes. The GPC undergoes co- and post-translational processing by the SKI-1/S1P protease to yield GP1, GP2, and the stable signal peptide (SSP), which form the mature glycoprotein complexes that are incorporated into virions to mediate cell attachment and entry. NP is the most abundantly present viral protein, and is the main structural component of the ribonucleoprotein (RNP) complex that is responsible for replication and genome transcription. The L segment (~7.2 kb) encodes both the RNA-dependent RNA polymerase (LP) and the matrix protein (Z), a small RING finger protein (8).

AHF is only arenavirus disease that can be prevented by vaccination. Candid #1 (Can) is a live attenuated vaccine strain derived from the human-isolated XJ strain that is used to manufacture vaccine in Argentina. The original XJ strain of Junin virus was serially passage through mouse brains, followed by cell culture passaging in fetal rhesus monkey lung (FRhL-2) cells to create the Can vaccine (9). The genetic differences between the wild-type JUNV and the Can vaccine have been studied in the past, and we and others (10) have previously determined that the primary differences that contribute to attenuation exist within the GPC (11). Moreover, between the 13^th^ mouse brain passage strain (XJ13) and the 44^th^ mouse brain passage strain (XJ44), the virus lost an N-linked glycosylation motif within the GP1 subunit of GPC. This resulted in a change in the glycosylation profile of the GPC, and this missing N-linked glycan causes the protein to trigger the unfolded protein response (UPR) within the endoplasmic reticulum (ER) of infected cells. This process induces ER stress, and an accumulation of improperly folded GPC within the ER, which leads to aggregation and degradation of the GPC through autophagy. The resulting virions contain a lower concentration of glycoprotein on the surface than the wild-type virions (12) without any obvious impact on virus growth in laboratory cells.

A single amino acid substitution within the transmembrane region of Can G2 significantly reduces neurovirulence of JUNV in mice and guinea pigs (13, 14). However, an acute, non-lethal visceral disease is still present in Hartley guinea pigs (11). Additionally, this substitution occurred during FRhL-2 passages and cannot account for the attenuated profiles of XJ13 and XJ44 intermediate strains (6). Here, we present evidence that the T168A amino acid substitution that eliminates an N-linked glycosylation motif within Can is significant for attenuation of both the visceral and neurological disease, and additionally improves the immunogenicity of the virus. However, the single nucleotide substitution that results in the T168A amino acid substitution is unstable and reverts to the wild-type sequence easily. The XJ13-specific substitutions within the GP1 subunit attenuate the visceral disease while preserving the virus neurovirulence.

## 2 Materials and methods

### 2.1 Virus propagation and titration

All recombinant viruses (rRom, rRom/GPC-XJ13, rRom/GPC-XJ44, rRom/GPC-T168A, and rRom/GPC-R484G) were rescued using a Pol-I/Pol-II driven rescue system described previously (13). Each virus clone was propagated in VeroE6 cells to generate a working stock using Dulbecco’s Modified Eagle Medium (DMEM) containing 5% fetal bovine serum (FBS). The virus working stocks were collected from the supernatant on day 4 post-infection, and the supernatant was passed through a 0.45µM filter for purification. Infectious particle concentrations were determined by plaque assay using Vero cells seeded into 12-well plates. Confluent monolayers were incubated with serial dilutions of virus stock for 1 hour, and then an overlay of 0.6% tragacanth gum and Modified Eagle Medium (2% FBS) was added. The monolayers were incubated at 37°C for 5 days, and then were fixed with 10% formalin for 30 minutes and stained with 0.25% crystal violet in 10% formalin. Individual plaques were counted to determine infectious particle titer. Each virus clone’s replication kinetics has been characterized previously *in vitro* (12).

### 2.2 Animal experiments

All animal studies have been reviewed and approved by the UTMB Animal Care and Use Committee (Protocol #1902007A, approved February 1, 2022) and were performed according to the National Institutes of Health guidelines for BSL-4 conditions. Six-week-old female Hartley guinea pigs were purchased from Charles River Laboratories and housed for seven days for acclimatization. Temperatures were recorded using DAS-8027-IUS readers and IPTT-300 transponders. Animals were anesthetized with isoflurane using a precision variable-bypass vaporizer to implant the transponder, and monitored for three days prior to inoculation of virus. Guinea pigs were anesthetized in the same manner, and inoculated with 10^3^ PFU rJUNV via the intraperitoneal route (i.p.) on day 0. For infections, 100uL of virus stock diluted in sterile PBS was given to each animal. Temperature was monitored telemetrically throughout the study and weight was recorded daily. Recording of death and disease symptoms was performed using the following definitions: discoordination, ataxia, transient seizures, or paralysis (hemiplegic or quadriplegic with the inability to reach the feeder or water bottle). Guinea pigs 3, 9, and 13 were required to be removed from the study prior to infection (veterinarian decision based on findings during daily wellness checks).

### 2.3 Hematology and clinical chemistry

Guinea pigs were bled via the vena cava at the time of euthanasia. Blood was collected into EDTA tubes and analyzed using an Abaxis Vetscan HM5 for hematology. Analyses were run at the time of collection. To obtain serum, whole blood was centrifuged at 2000 x g for 15 minutes in BD Microtainer serum separator tubes. Clinical chemical analysis was performed using serum collected at time of euthanasia with an Abaxis Vetscan VS2 using the comprehensive diagnostic profile following the manufacturer’s instructions.

### 2.4 Histopathology

Tissue samples were collected from each guinea pig at necropsy. Tissue samples were fixed in 10% buffered formalin for one week, and the formalin was drained and replaced 3 times (one change every 48 hours) during this process. Tissues were transferred to a 70% ethanol solution. After being stored in 70% ethanol for 12 hours, the tissues were processed using a Tissue-Tek VIP 6 AI Vacuum Infiltration Processor (Sakura Finetek USA, Inc., Torrance, CA), paraffin-embedded, sectioned at 5 µm using a HM 325 Rotary Microtome (Thermo Fisher Scientific, Inc., Waltham, MA), and mounted on slides. Hematoxylin and eosin (H&E) staining, dehydration, and coverslip placement were conducted using a Tissue-Tek Prisma Plus Automated Slide Stainer (Sakura Finetek USA, Inc., Torrance, CA). All processing was performed in a CAP-accredited laboratory at UTMB by licensed histotechnologists. Pathology scoring was performed blinded, and arbitrary values (1-4) were assigned based on the severity of finding.

### 2.5 Plaque reduction neutralization assays

Serum from surviving guinea pigs was 2x serially diluted in PBS. A working stock of virus was diluted in DMEM to a final concentration of 600 PFU/mL., and 100µL of each serum dilution was combined with 100µL of virus working stock. The mixtures were incubated at 37°C for 1 hour in 96-well plates, rocking gently. After incubation, 100µL of each mixture was added to a well of a 6-well plate of Vero cells that had been grown to confluence. The 6-well plates were incubated for another hour at 37°C, and 2mL of overlay (0.6% tragacanth gum and Modified Eagle Medium containing 2% FBS) was added. The plates were incubated for 5 days and fixed with 10% formalin for 30 minutes, followed by staining with 0.25% crystal violet in 10% formalin.

## 3 Results

### 3.1 Multiple amino acid substitutions within the GP1 subunit contribute to the attenuation of Can

**Figure 1:**
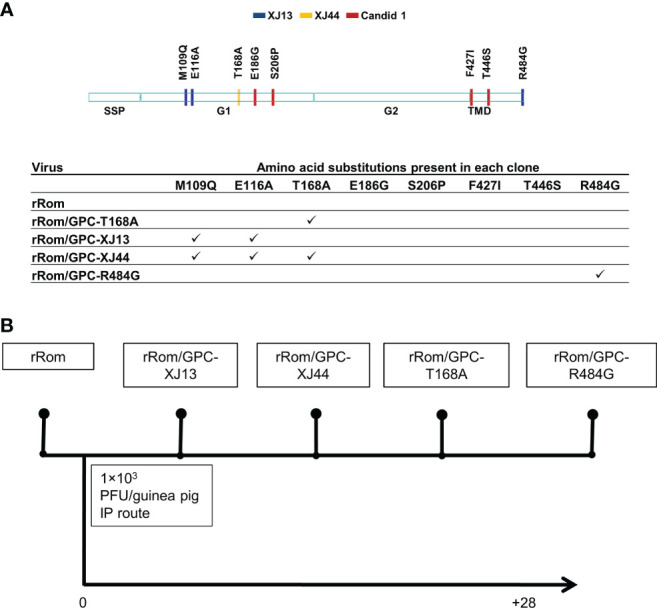
Schematic representation of recombinant viruses and experimental design. **(A)** The provided schematic indicates all of the amino acid differences between Rom and Can GPC. The table indicates which amino acid differences are present in each clone. **(B)** Guinea pigs were infected with recombinant viruses at 10^3^ PFU intraperitoneally. Guinea pigs were monitored for 28 days post-infection, and survivors were euthanized for necropsy.

**Figure 2:**
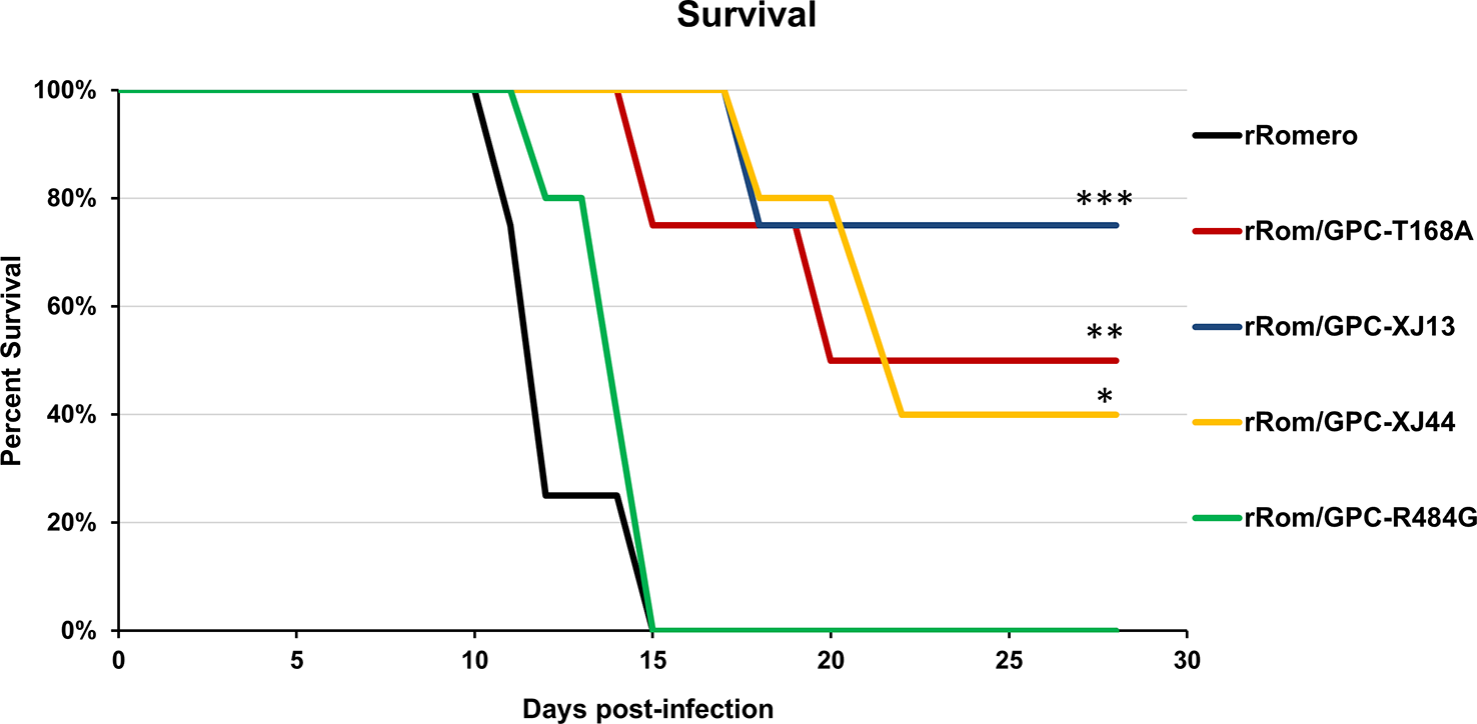
Group survival. Group survival percentages over the course of infection are shown. Statistics were performed using a one-sided t-test. ***P=0.01, **P=0.05, *P=0.09. There is no statistically significant difference between XJ13, XJ44, and T168A groups.

**Figure 3:**
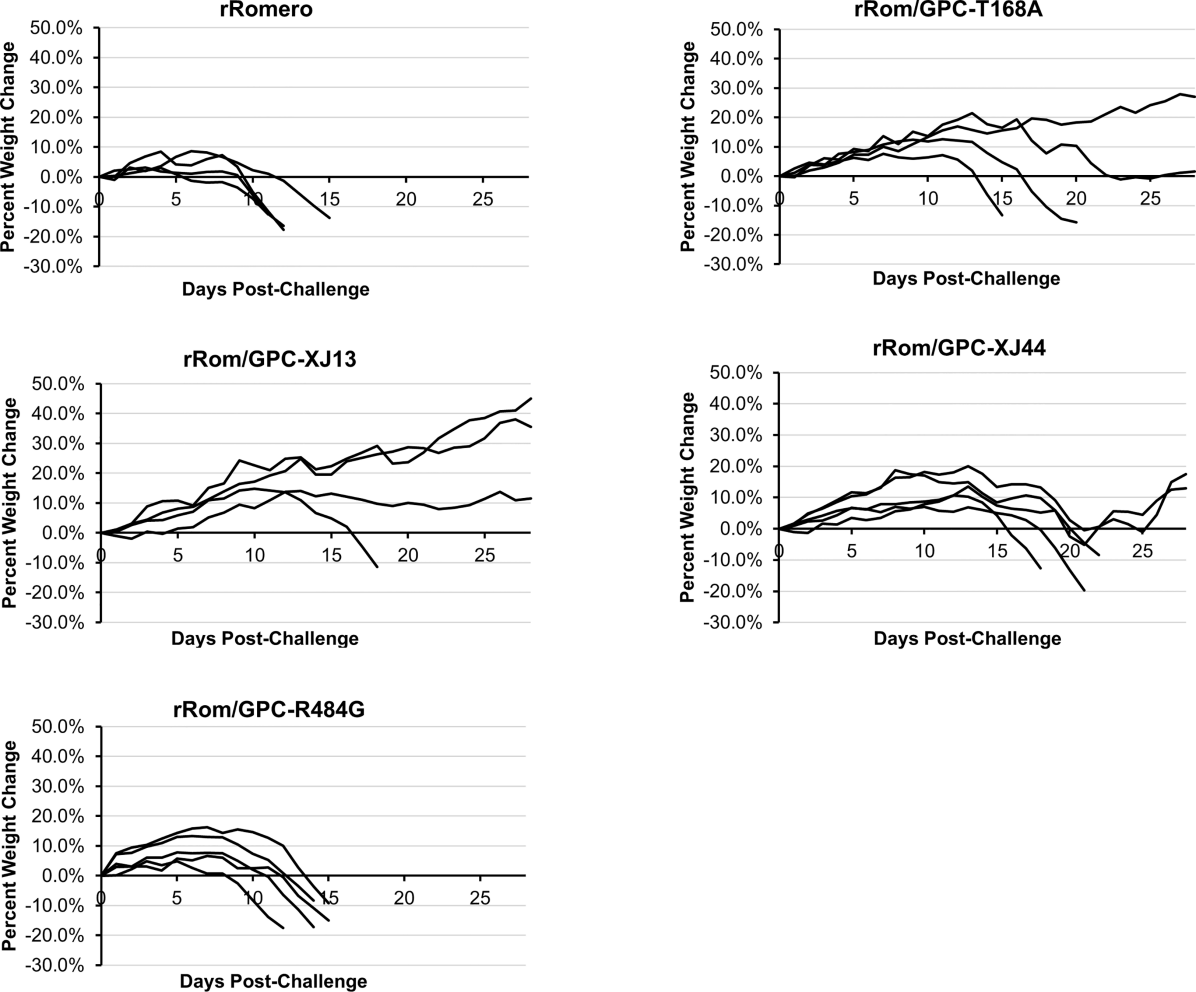
Group weight change. Guinea pigs were individually weighed throughout the course of the infection. Weights are provided as a percentage change from their starting weight on the day of challenge. Guinea pigs losing more than 15 percent of their starting weight were humanely euthanized as a study endpoint. Each line represents a single guinea pig within the group.

**Figure 4:**
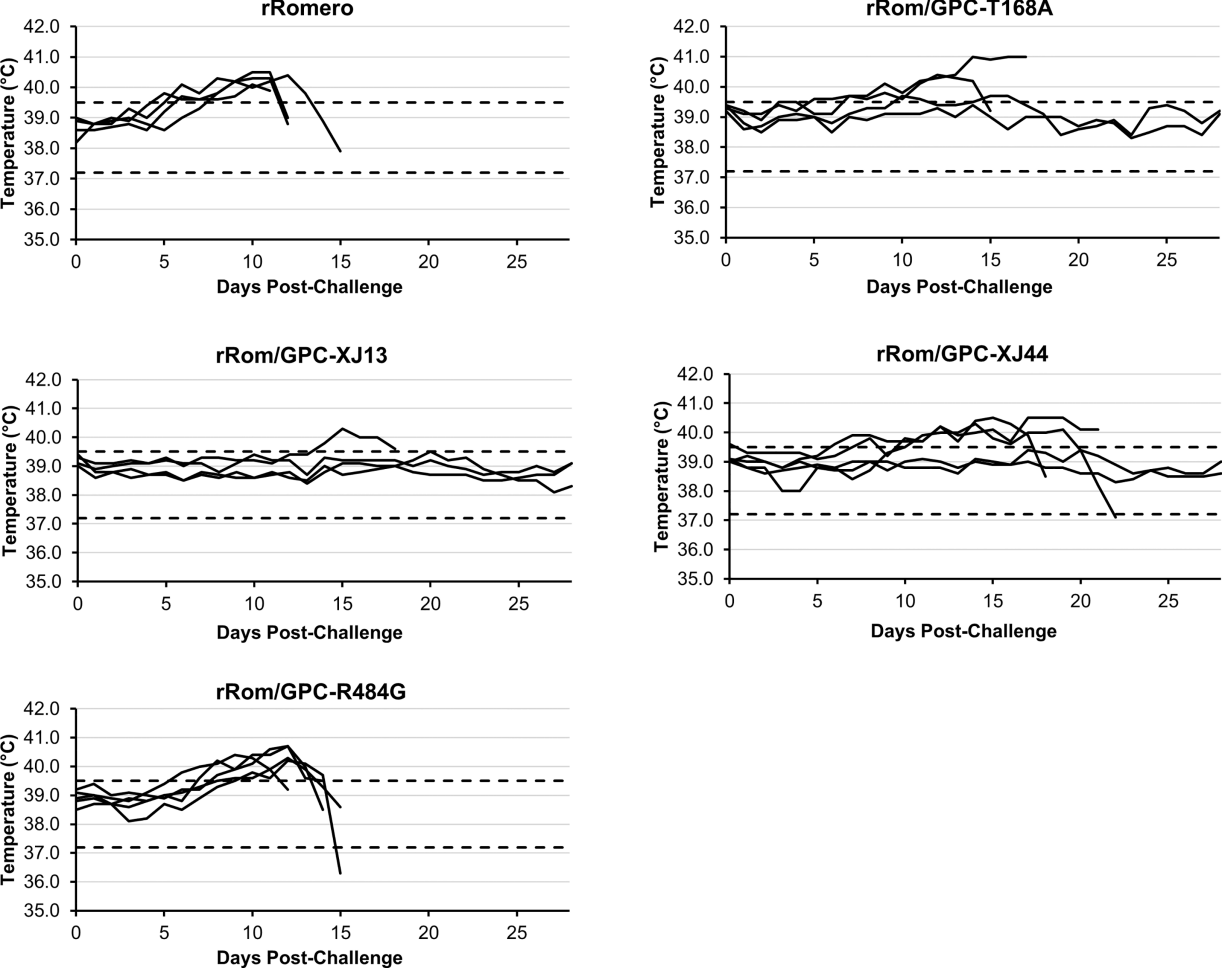
Group temperatures. Guinea pigs were implanted with an IPTT-300 transponder subcutaneously, and temperatures were measured daily to detect hyperthermia or hypothermia. Dotted lines indicate the upper and lower limits of normal values. Each line represents a single guinea pig within the group.

**Figure 5:**
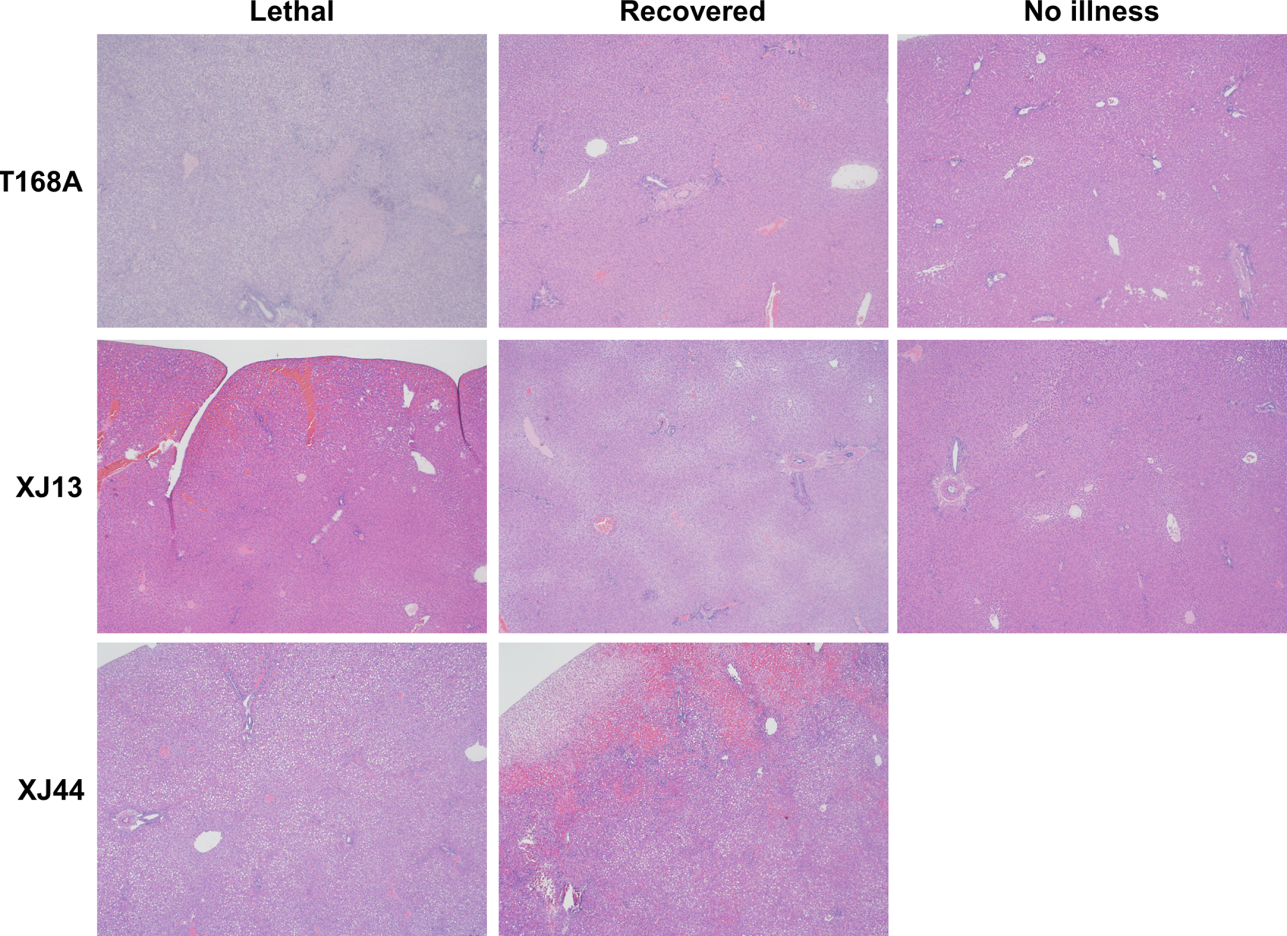
Comparison of pathological findings between representative guinea pigs from separate groups. Representative liver histology images for each clinical outcome are provided for T168A, XJ13, and XJ44-specific groups. Images represent lethal illness, recoveries, or guinea pigs with no detectable illness.

We have previously shown that the Can GPC is the primary gene responsible for attenuation in Hartley guinea pigs (11) and that the XJ44-specific T168A substitution results in ER stress and degradation of the JUNV GPC (12). While the F427I transmembrane substitution attenuates neurovirulence, the substitution does not completely prevent visceral disease (11, 13, 14). Therefore, additional mutations must play a role in Can attenuation, either in the ectodomain or the cytoplasmic tail. Based on our *in vitro* data (12), we aimed to determine what role the T168A substitution plays in attenuation *in vivo*. Due to the lack of availability of the XJ strain, we have previously generated a recombinant JUNV Romero strain (Rom), and additional clones that contain GPCs from key passages in the attenuation process, specifically XJ13 (rRom/GPC-XJ13) and XJ44 (rRom/GPC-XJ44). We have also created a Rom clone containing only the T168A substitution (rRom/GPC-T168A) (12). Additionally, to rule out the involvement of the R484G substitution within the cytoplasmic tail domain of GPC as an attenuating factor, we also created a Rom clone expressing only the R484G substitution (rRom/GPC-R484G). Accordingly, we challenged groups of Hartley guinea pigs (i.p., 10^3^ PFU) with rRom, rRom/GPC-XJ13, rRom/GPC-XJ44, rRom/GPC-T168A, or rRom/GPC-R484G and monitored their survival and disease progression (**Figure 1**). Both rRom and rRom/GPC-R484G behaved like wild type virus and were completely lethal with the exception of GP3, which had no sign of infection (**Figure 2**). Guinea pigs infected with rRom developed a fever and steady weight loss starting at 7 days post-infection (**Figures 3**, **4**). The rRom/GPC-R484G group progressed identically, suggesting that this substitution plays no role in attenuation alone. Three guinea pigs infected with rRom/GPC-XJ13 developed no detectable illness, while a single guinea pig experienced a delayed fever and weight loss, with an onset at 10 days post-infection. Guinea pigs within both the rRom/GPC-XJ44 and the rRom/GPC-T168A groups had a delayed onset of symptoms, developing fever and weight loss at day 13 post-infection. We were able to isolate the virus RNA from the livers of 3 guinea pigs (GP 6, 7, 18) from the XJ44 and T168A groups (**Figure 5**) at the study endpoint and sequence the entire viral genomes. All of the virus sequences revealed a single amino acid reversion that restores the N-linked glycosylation motif in the GPC, with no amino acid substitutions present in other genes. Interestingly, two substitutions were A168S (GP 6, 18) instead of the original A168T reversion, suggesting that the glycosylation motif (N-X-S/T), and not the specific Thr residue, is responsible for the virulent phenotype.

The rRom and rRom/GPC-R484G groups both developed thrombocytopenia and immunosuppression as shown by a decrease in platelets, lymphocytes, and total white blood cells in all guinea pigs that succumbed to infection (**Table 1**). Alanine aminotransferase (ALT) levels were elevated in 3 of the 4 Rom-infected guinea pigs and in all of the rRom/GPC-R484G guinea pigs. ALT levels were elevated in two guinea pigs in the rRom/GPC-T168A group and in one guinea pig in the rRom/GPC-XJ44 group. All guinea pigs in the rRom/GPC-XJ13 group were within normal ALT ranges (**Table 1**). An increase in ALT levels indicates acute damage to hepatocytes in guinea pigs and develops during the acute phase of AHF.

**Table 1:** Blood chemistry and hematology values. Normal ranges are listed in the column headings.

		Post-inoculation values					
Group	Guinea Pig	White Blood Cell Count (k/uL) (4.5-11.0)	Lymphocyte Count (k/uL) (1.5-10.6)	Platelet Count (10^3/mL) (193-427)	Alanine Aminotransferase (U/L) (19-39)	Amylase (U/L) (850-1380)	Albumin (g/dL) (2.7-3.3)
**rRom**	1	1.9	1.85	9	94	1111	2.3
	2	1.6	1.45	14	102	1175	3.1
	4	1.74	1	12	133	1053	3.3
	5	1.25	1.08	11	85	890	2.5
**rRom/GPC-T168A**	6	2.1	0.7	6	60	1064	3.4
	7	1.69	0.49	25	83	1776	3.6
	8	4.68	2.99	182	23	1347	4.3
	10	6.67	2.62	253	31	1088	4.5
**rRom/GPC-XJ13**	11	11.99	5.52	366	28	1130	4.1
	12	7.21	2.3	379	29	1179	4.7
	14	5.21	0.85	298	29	1140	5
	15	8.11	2.97	277	41	967	4.2
**rRom/GPC-XJ44**	16	7.09	3.01	68	46	1333	3.5
	17	14.1	12.01	168	28	1058	4.2
	18	2.53	0.51	45	80	1487	3.9
	19	4.14	3.02	296	21	989	4.4
	20	1.74	0.74	42	38	1325	3.3
**rRom/GPC-R484G**	21	2.55	2.48	3	92	1214	2.7
	23	2.42	2.38	10	76	1102	2.6
	24	1.5	1.47	8	62	1056	2.5
	25	2.04	1.97	7	79	1236	2.1

**Figure 6:**
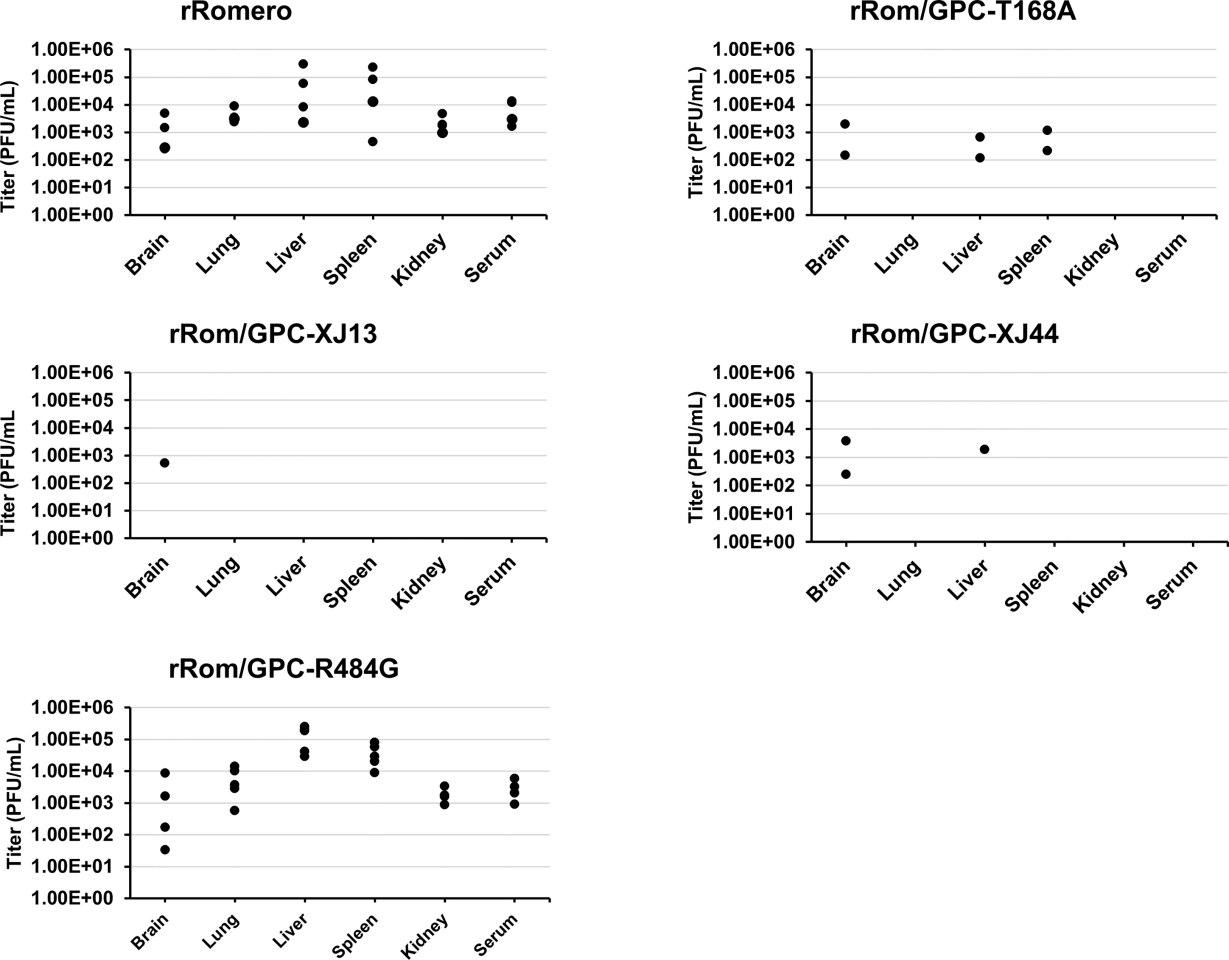
Organ titers. Organ titers were measured through plaque assay following tissue homogenization. Individual organ titers are represented as single points for each respective organ. The detection limit is 100 particles per mL of homogenized tissue.

We detected a high viral load in the brain, lungs, liver, spleen, and kidney in the rRom and rRom/GPC-R484G groups, as well as viremia (**Figure 6**). In the rRom/GPC-XJ13 group, only guinea pig 14 had a detectable viral load in the brain and this animal developed a hind limb paralysis. Similarly, the rRom/GPC-XJ44 had two guinea pigs which succumbed to illness due to hind limb paralysis. Each of the guinea pigs had a high viral load in the brain without a detectable infectious virus in other organs (**Figure 6**). Only a single lethal infection within the rRom/GPC-XJ44 group was caused by visceral disease, and virus was isolated only from the liver. Within the rRom/GPC-T168A group, we could detect virus in the brain, liver, and spleen in guinea pigs that succumbed to illness (**Figure 6**). There were no instances of hind limb paralysis in this group.

After collecting serum from each of the surviving guinea pigs in each group, we performed assays to measure the homologous PRNT80 values for each individual. Guinea pigs surviving the rRom/GPC-XJ13 infection produced neutralizing antibodies with a 1:20 or 1:40 PRNT80 value. The guinea pigs from the rRom/GPC-XJ44 group exhibited a slight increase in neutralizing antibody titers, with values of 1:80. The rRom/GPC-T168A had the highest PRNT80 values, with the two survivors measuring either 1:80 or 1:160 (**Table 2**). Measuring heterologous titers against rRom provided a similar result, with the rRom/GPC-T168A and rRom/GPC-XJ44 groups having the highest neutralizing titers against rRom (**Table 2**).

**Table 2:** PRNT80 values were obtained for either homologous strains, or against Romero.

Group	Guinea Pig	PRNT80 (homologous)	PRNT80 (heterologous [Rom])
rRom/GPC-T168A	8	1:160	1:40
	10	1:80	1:40
rRom/GPC-XJ13	11	1:20	<1:10
	12	1:40	<1:10
	15	1:40	<1:10
rRom/GPC-XJ44	17	1:80	1:20
	19	1:80	1:40

The detection limit is a 1:10 dilution of serum. A t-test was performed to calculate statistical significance between T168A and XJ13 (P=0.038 homologous, P=0.037 heterologous), T168A and XJ44 (P=0.16 homologous, P=0.422 heterologous), and XJ13 and XJ44 (P=0.037 homologous, P=0.037 heterologous).

### 3.2 Pathology

Guinea pigs with fever and weight loss each exhibited confluent necrosis within the liver (**Figure 5**). Foamy macrophages were observed in many animals with confluent necrosis, except for guinea pig 1 in the rRom group, guinea pig 20 in the XJ44 group, and guinea pig 25 in the R484G group (**Table 3**). All guinea pigs exhibited lobular inflammation and portal inflammation, often with mild interface activity. We observed the presence of lipid droplets in hepatocytes in almost all guinea pigs with lethal outcomes (**Figure 5**, **Table 3**), the exceptions being guinea pigs 9 and 14 in the T168A and XJ13 groups, respectively.

**Table 3:** Liver pathology scoring.

			TOTAL	Interface activity	Confluent necrosis	Lobular inflammation/apoptosis	Portal inflammation	Foamy macrophages	Lipid droplets
Group	GP#	Clinical outcome	n/49	1-4+	1-4+	1-4+	1-4+	1-3+	1-3+
**Romero**	1	Lethal	7	1	1 (focal)	2	1	0	2
	2	Lethal	6	1	0	2	1	0	2
	4	Lethal	11	1	1-2+	2	1	3	2
	5	Lethal	6	1	0	2	1	0	2
**rRom/GPC-T168A**	6	Lethal	11	1	2 (focal patch)	2	1	2	3
	7	Lethal	6	1	0	2	1	0	3
	8	Recovered	3	1	0	1	1	0	0
	10	No illness	3	1	0	1	1	0	0
**rRom/GPC-XJ13**	11	No illness	3.5	0	0	3	0.5	0	0
	12	Recovered	3.5	0	0	3	0.5	0	0
	14	Lethal	4	1	0	2	1	0	0
	15	No illness	4	1	0	2	1	0	0
**rRom/GPC-XJ44**	16	Lethal	11	1	3	2	1	1	3
	17	Recovered	3	1	0	1	1	0	0
	18	Lethal	12	1	4	2	1	2	2
	19	Recovered	1.5	0	0	1	0.5	0	0
	20	Lethal	6	0	1	2	2	0	1
**rRom/GPC-R484G**	21	Lethal	4	1	0	1	1	0	1
	22	Lethal	9	1	2	2	1	1	2
	23	Lethal	6	1	0	1	1	0	3
	24	Lethal	5	1	0	1	1	0	2
	25	Lethal	6	0	3	1	1	0	1

Values of 1-4 were assigned based on prominence of each finding.

Within the brain, guinea pigs in the rRom group, as well as guinea pig 9 in the T168A group, guinea pig 11 in the XJ13 group, and guinea pig 21 in the R484G group exhibited a normal histology. We noticed encephalitis primarily localized to the brainstem in each of the guinea pigs within the R484G group, with the exception of guinea pig 21, as well as in guinea pigs 6, 7, and 10 in the T168A group, and guinea pig 15 in the XJ13 group (**Table 4**). In some guinea pigs, the observed encephalitis extended into the striatum (guinea pig 12 in the XJ13 group, guinea pigs 16-18 in the XJ44 group) and even into the neocortex (guinea pig 8 in the T168A group, guinea pig 14 in the XJ13 group, guinea pigs 19 and 20 in the XJ44 group).

**Table 4:** Brain pathology scoring.

Group	Guinea pig ID#	Brain Pathology Score	Hind Limb Paralysis
Rom	1	0	
	2	0	
	4	0	
	5	0	
Rom/GPC-T168A	6	1	
	7	1	
	8	3	
	9	0	
	10	1	
Rom/GPC-XJ13	11	0	
	12	2	
	14	3	Yes
	15	1	
Rom//GPC-XJ44	16	2	Yes
	17	2	
	18	2	
	19	3	
	20	3	Yes
Rom/GPC-R484G	21	0	
	22	1	
	23	1	
	24	1	
	25	1	

Scoring: 0 - normal/congestion, 1 - mild pathology: encephalitis mainly in the brainstem, 2 - moderate pathology: encephalitis extends into striatum and/or dentate nucleus of cerebellum, 3 - severe pathology: further involves neocortex" after 2 - moderate pathology: encephalitis extends into striatum and/or dentate nucleus of cerebellum.

Values are further described in the table. Guinea pigs in which hind limb paralysis was observed are indicated in the table.

Values of 1-4 were assigned based on histological findings.

## 4 Discussion

Previously, we have shown that the primary gene responsible for Can attenuation is the GPC gene (11). However, it is not the sole contributing factor for Can attenuation. We also demonstrated that a mutation in the RING domain of Can Z contributes to attenuation in the guinea pig model (15). While the F427I mutation within the transmembrane domain of JUNV GPC was shown to be attenuating in suckling mice, the mutation was only partially attenuating in the guinea pig model (11). Recently, we discovered that a T168A amino acid substitution within Can GPC is located within an N-linked glycosylation motif, resulting in the absence of a glycan at N166. Due to the heavy involvement of glycans in glycoprotein folding, trafficking, cleavage, and immune escape, the effect of this substitution on virulence was warranted. Removal of specific N-linked glycosylation motifs has previously been shown to be attenuating in both Rabies virus and H1N1 influenza (16–18). While the exact mechanism for attenuation has not been resolved for either Rabies or H1N1, we have shown that the T168A mutation in Can triggers accumulation of GPC within the ER, resulting in ER stress and degradation of the protein (12). However, this mechanism has not previously been linked to attenuation of Can. Here we have shown that the N-linked glycan at amino acid position N166 is important for JUNV virulence and dissemination, providing an attenuated profile when the glycan is removed. While protein folding and trafficking likely play a large role in the attenuation, the T168A substitution may also contribute to a decreased immune evasion as well. The T168A-infected group produced the highest neutralizing antibody titer among all survivors (**Table 2**). Determining whether the absence of this glycan provides a mechanism of immune evasion should be tested in the future.

While the vast majority of amino acid differences between Rom and Can GPC exist within the ectodomain, one difference exists within the transmembrane domain of GP2, while a second difference is present within the cytoplasmic tail of GP2. Multiple studies in both mice and guinea pigs have shown a partial attenuation due to the F427I transmembrane substitution (11, 14), we also needed to investigate whether the R484G substitution would play a role in the attenuation of rRom. By all measures in this study, the rRom/GPC-R484G virus performed similarly to the rRom virus and wild type. The average time to death, viral load, hematology, and pathology were similar in all infected guinea pigs. While this substitution alone does not appear to impact the pathogenesis or severity of illness, it is possible that the substitution was an adaptation in response to mutations within the Z protein of XJ13 and Can. The presence of the R484G substitution within the GPC could perform a compensatory role to restore the interaction between Z and GPC. Potential interactions between the R484G substitution and the V18A or V64G substitutions within Z need further investigation.

Within the rRom/GPC-XJ44 and rRom/GPC-T168A groups, only guinea pig 10 had no detectable illness. Each of the guinea pigs that became ill within these groups had a delayed onset of illness and the visceral disease was similar to the disease observed in the rRom-infected guinea pigs. While the disease in the T168A group was primarily visceral disease, guinea pigs within the XJ44 group had a neurological disease as well. Disease within the rRom/GPC-XJ13 was primarily neurological. Interestingly, XJ13 and XJ44 were derived through mouse brain passaging (9), where the virus potentially adapted to mouse brain tissue. In the instance in which rRom/GPC-XJ13 became lethal, it was due to a hind limb paralysis in which the only detectable virus could be found in the brain. Similarly, two of the three lethal rRom/GPC-XJ44 infections were due to hind limb paralysis in which the virus was localized to the brain. After we sequenced the virus, we found that in both cases, the T168A substitution had reverted back to a wild-type phenotype in which the glycosylation motif had been restored. This created an XJ13-like GPC after the reversion, reproducing the neurological illness with no detectable visceral disease that we observed within the XJ13-infected group. Therefore, it appears that the visceral illness is attenuated with the presence of XJ13-specific amino acid substitutions, but the neurological illness is still present in some cases. This phenomenon has been observed previously in a chimeric Yellow fever/Japanese encephalitis live attenuated vaccine. A reversion at position 279 of the envelope protein resulted in an increased neurovirulence, but a decreased viscerotropism (19).

In each lethal infection within the rRom/GPC-T168A group, we detected a reversion at the 168 position back to either a Ser or Thr residue, which restores the glycosylation motif. Interestingly, we observed a delayed disease progression in each of the guinea pigs with reversions. While the T168A substitution does seem to provide a completely attenuated profile in the absence of reversion, it is likely that a reversion occurs due to the instability of the single nucleotide mutation. The T168A substitution occurred at passage 17 in mouse brain tissue, and was retained through XJ44 (9). This single amino acid substitution appears to be stable in brain tissue, but unstable by itself in visceral tissue. During the passaging in FRhL cells, the GPC acquired four additional amino acid substitutions. One or more of these substitutions could be stabilizing the T168A substitution in visceral tissue. However, we cannot rule out the possibility that a substitution within another gene is providing stabilization. Further investigation would be needed to determine whether this is in fact the case. The presence of multiple attenuating mutations within the Can vaccine (14, 15) likely drastically decreases the chance of a reversion to a virulent phenotype, as each mutation is either partially or fully attenuating independently of other mutations.

In this study, we have determined that the T168A substitution is attenuating in rRom GPC, however the mutation is highly unstable. Additionally, the XJ13-specific amino acid substitutions are partially attenuating in the rRom backbone, causing only neurological disease with an absence of visceral disease. Due to the presence of multiple attenuating mutations within Can, the reversion of any individual amino acid does not result in a reversion to a pathogenic phenotype. In combination, the presence of multiple mutations within Can provide a very stable attenuated phenotype. However, in order to rationally design live attenuated vaccine candidates, the potentially unstable nature of mutations within glycosylation motifs will need to be considered. This data provides supporting evidence that targeting the N-linked glycosylation motifs of arenavirus glycoproteins is a viable strategy for the design of live attenuated vaccine candidates to combat arenavirus-associated illness.

## Data availability statement

The raw data supporting the conclusions of this article will be made available by the authors, without undue reservation.

## Ethics statement

The animal study was reviewed and approved by Institutional Animal Care and Use Committee (IACUC), University of Texas Medical Branch.

## Author contributions

JTM, JM, RS, TW, and EM were involved in conducting guinea pig studies and in data collection. TW, HS-L, B-HP conducted histology. JTM, JM, TW, CH, and SP were involved in writing and editing the manuscript and figures. All authors contributed to the article and approved the submitted version.
